# Bis(2,6-diamino-3,5-dibromo­pyridinium) hexa­bromidostannate(IV)

**DOI:** 10.1107/S1600536809015189

**Published:** 2009-04-30

**Authors:** Rawhi H. Al-Far, Salim F. Haddad, Basem Fares Ali

**Affiliations:** a Faculty of Information Technology and Science, Al-Balqa’a Applied University, Salt, Jordan; b Department of Chemistry, University of Jordan, Amman, Jordan; c Department of Chemistry, Al al-Bayt University, Mafraq 25113, Jordan

## Abstract

The asymmetric unit of the title compound, (C_5_H_6_Br_2_N_3_)_2_[SnBr_6_], contains one cation and one half-anion in which the Sn atom is located on a crystallographic centre of inversion and is in a quasi-octa­hedral geometry. The crystal structure is assembled *via* hydrogen-bonding inter­actions of two kinds, N(pyridine/amine)—H⋯Br—Sn, along with C—Br⋯Br—Sn interactions [3.4925 (19) Å]. The cations are involved in π–π stacking, which adds an extra supra­molecularity as it presents a strong case of offset-face-to-face motifs [centroid–centroid distance = 3.577 (3) Å]. The inter­molecular hydrogen bonds, short Br⋯Br inter­actions and π–π stacking result in the formation of a three-dimensional supra­molecular architecture.

## Related literature

For general background to hybrid organic–inorganic compounds, see: Aruta *et al.* (2005 ▶); Hill (1998 ▶); Kagan *et al.* (1999 ▶); Knutson *et al.* (2005 ▶); Raptopoulou *et al.* (2002 ▶). For related structures, see: Al-Far & Ali (2007 ▶); Al-Far, Ali & Al-Sou’od (2007 ▶); Ali & Al-Far (2007 ▶); Ali *et al.* (2008 ▶); Ali, Al-Far & Ng (2007 ▶); Awwadi *et al.* (2007 ▶); Tudela & Khan (1991 ▶); Willey *et al.* (1998 ▶). For bond-length data, see: Allen *et al.* (1987 ▶).
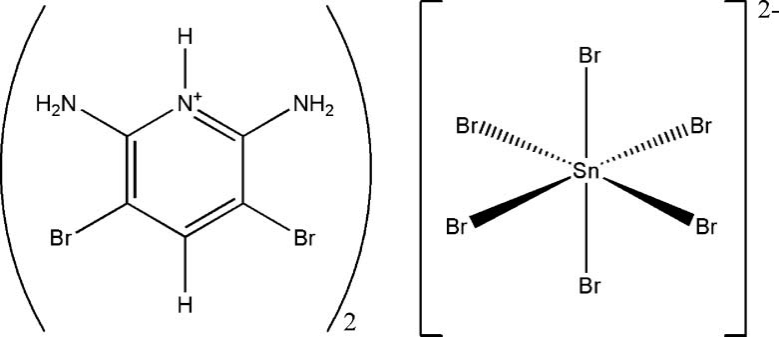


## Experimental

### 

#### Crystal data

(C_5_H_6_Br_2_N_3_)_2_[SnBr_6_]
*M* *_r_* = 1133.97
Monoclinic, 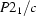

*a* = 8.3696 (14) Å
*b* = 16.720 (2) Å
*c* = 9.5814 (15) Å
β = 112.556 (12)°
*V* = 1238.3 (3) Å^3^
*Z* = 2
Mo *K*α radiation
μ = 17.18 mm^−1^
*T* = 295 K
0.30 × 0.30 × 0.20 mm

#### Data collection

Bruker P4 diffractometer
Absorption correction: ψ scan (*XSCANS*; Bruker, 1996 ▶) *T*_min_ = 0.007, *T*_max_ = 0.035
2825 measured reflections
2162 independent reflections
1437 reflections with *I* > 2σ(*I*)
*R*_int_ = 0.078
3 standard reflections every 97 reflections intensity decay: 0.01%

#### Refinement

*R*[*F* ^2^ > 2σ(*F* ^2^)] = 0.059
*wR*(*F* ^2^) = 0.146
*S* = 1.00
2162 reflections
124 parameters
H-atom parameters constrained
Δρ_max_ = 0.97 e Å^−3^
Δρ_min_ = −1.63 e Å^−3^

### 

Data collection: *XSCANS* (Bruker, 1996 ▶); cell refinement: *XSCANS*; data reduction: *SHELXTL* (Sheldrick, 2008 ▶); program(s) used to solve structure: *SHELXS97* (Sheldrick, 2008 ▶); program(s) used to refine structure: *SHELXL97* (Sheldrick, 2008 ▶); molecular graphics: *SHELXTL*; software used to prepare material for publication: *SHELXTL*.

## Supplementary Material

Crystal structure: contains datablocks I, global. DOI: 10.1107/S1600536809015189/hk2669sup1.cif

Structure factors: contains datablocks I. DOI: 10.1107/S1600536809015189/hk2669Isup2.hkl

Additional supplementary materials:  crystallographic information; 3D view; checkCIF report

## Figures and Tables

**Table 1:** Selected geometric parameters (Å, °)

Sn1—Br1	2.6002 (13)
Sn1—Br2	2.5768 (14)
Sn1—Br3	2.6131 (14)

Symmetry code: (i) 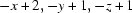.

**Table 2:** Hydrogen-bond geometry (Å, °)

*D*—H⋯*A*	*D*—H	H⋯*A*	*D*⋯*A*	*D*—H⋯*A*
N1—H1⋯Br3^ii^	0.86	2.54	3.354 (9)	159
N2—H2*B*⋯Br3^ii^	0.86	2.88	3.612 (12)	144
N3—H3*A*⋯Br2^ii^	0.86	2.79	3.608 (10)	160
N3—H3*B*⋯Br1^iii^	0.86	2.82	3.604 (10)	153

Symmetry codes: (ii) 
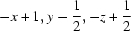
; (iii) 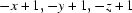.

**Fig. 1.**
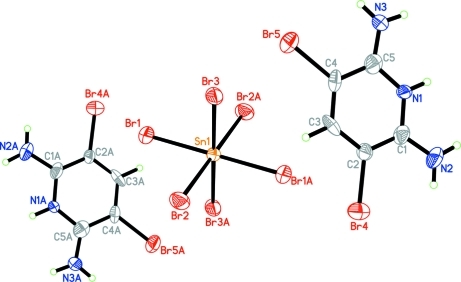
The molecular structure of the title molecule, with the atom-numbering scheme. Displacement ellipsoids are drawn at the 50% probability level [symmetry code (A): 2 - x, 1 - y, 1 - z].

**Fig. 2.**
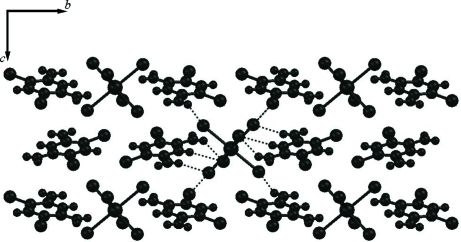
A partial packing diagram of the title compound. Hydrogen bonds and Br···Br interactions are shown as dashed lines.

## supplementary crystallographic information

### Comment

Non-covalent interactions play an important role in organizing structural units in both natural and artificial systems. Hybrid organic-inorganic compounds are of great interest owing to their ionic, electrical, magnetic and optical properties (Hill, 1998; Kagan *et al.*, 1999; Raptopoulou *et al.*, 2002). Tin metal-halo based hybrids are of particular interest as being materials with interesting optical and magnetic properties (Aruta *et al.*, 2005; Knutson *et al.*, 2005; Kagan *et al.*, 1999). We are currently carrying out studies about lattice including different types of intermolecular interactions (aryl···aryl, *X*···*X*, *X*···aryl and *X*···H). Within our research of hybrid compounds containing tin metal (Al-Far & Ali 2007; Al-Far, Ali & Al-Sou'od, 2007; Ali & Al-Far, 2007; Ali, Al-Far & Ng, 2007), we report herein the crystal structure of the title compound.

The asymmetric unit of the title compound contains one cation and one-half anion, in which the Sn atom is located on a crystallographic centre of inversion and is in a quasi-octahedral geometry (Fig. 1 and Table 1). The Sn-Br bonds are in accordance with the corresponding values in similar compounds (Willey *et al.*, 1998; Tudela & Khan 1991; Ali *et al.*,  2008; Al-Far & Ali 2007). The pyridine ring of the starting cation have undergone bromination during the synthesis process (Al-Far & Ali, 2007). In the cation, the bond lengths (Allen *et al.*,  1987) and angles are within normal ranges.

In the crystal structure, weak intermolecular N-H···Br interactions (Table 2) link the molecules into alternating layers of cations and stacks of anions (Fig. 2), in which they may be effective in the stabilization of the structure. The anion stacks are interacting with the cation layers in an extensive hydrogen bonding and Br···Br halogen bonding interactions. Each anion is surrounded by six cations *via* three H—N—H···Br, one N—H···Br interactions and the symmetry related ones along with one Br···Br interaction [Br2···Br4^i^ = 3.4925 (19) Å, symmetry code (i): 2 - x, 1 - y, -z)] and the symmetry related one. On the other hand, each cation is associated with three anions, through six (N_pyridinic_, N_aminic_)—H···Br—Sn hydrogen bonding interactions, and by one C—Br···Br—Sn interaction. It is noteworthy that structural and theoretical results (Awwadi *et al.* 2007; and references therein), show the significance of linear C—Br···Br synthons in influencing structures of crystalline materials and in use as potential building blocks in crystal engineering *via* supramolecular synthesis.

Moreover, interactions between cations perpendicular to [101] represent a case of strong offset face-to-face π–π motif. This is evident by the centroids separation distance of 5.059 (3) Å, with the perpendicular distance between planes being 3.577 (3) Å (the sliding angle between planes is 45.0 (3)°).

### Experimental

For the preparation of the title compound, the warm solution of SnCl_2_ metal (1.0 mmol) dissolved in absolute ethanol (15 ml) was mixed with a stirred hot solution of 2,6-diaminopyridine (98%; 2 mmol) dissolved in ethanol (20 ml). The mixture was acidified with HBr (48%, 2-3 ml), and then treated with liquid Br_2_ (2-3 ml). The resulting mixture was stirred for 3 h, and then allowed to evaporate at room temperature. The salt crystallized over 2 d, as nice parallelepiped yellow crystals (yield; 82%).

### Refinement

H atoms were positioned geometrically, with N-H = 0.86 Å (for NH and NH_2_) and C-H = 0.93 Å for aromatic H, respectively, and constrained to ride on their parent atoms, with U_iso_(H) = 1.2U_eq_(C,N).

### Crystal data

**Table:**

(C_5_H_6_Br_2_N_3_)_2_[SnBr_6_]	*F*(000) = 1028
*M**_r_* = 1133.97	*D*_x_ = 3.042 Mg m^−^^3^
Monoclinic, *P*2_1_/*c*	Mo *K*α radiation, λ = 0.71073 Å
Hall symbol: -P 2ybc	Cell parameters from 29 reflections
*a* = 8.3696 (14) Å	θ = 5.7–12.5°
*b* = 16.720 (2) Å	µ = 17.18 mm^−^^1^
*c* = 9.5814 (15) Å	*T* = 295 K
β = 112.556 (12)°	Parallelepiped, yellow
*V* = 1238.3 (3) Å^3^	0.30 × 0.30 × 0.20 mm
*Z* = 2	

### Data collection

**Table:**

Bruker P4 diffractometer	1437 reflections with *I* > 2σ(*I*)
Radiation source: fine-focus sealed tube	*R*_int_ = 0.078
graphite	θ_max_ = 25.0°, θ_min_ = 2.4°
ω scans	*h* = −9→1
Absorption correction: ψ scan (*XSCANS*; Bruker, 1996)	*k* = −19→1
*T*_min_ = 0.008, *T*_max_ = 0.035	*l* = −10→11
2825 measured reflections	3 standard reflections every 97 reflections
2162 independent reflections	intensity decay: 0.01%

### Refinement

**Table:**

Refinement on *F*^2^	Primary atom site location: structure-invariant direct methods
Least-squares matrix: full	Secondary atom site location: difference Fourier map
*R*[*F*^2^ > 2σ(*F*^2^)] = 0.059	Hydrogen site location: inferred from neighbouring sites
*wR*(*F*^2^) = 0.146	H-atom parameters constrained
*S* = 1.00	*w* = 1/[σ^2^(*F*_o_^2^) + (0.0766*P*)^2^] where *P* = (*F*_o_^2^ + 2*F*_c_^2^)/3
2162 reflections	(Δ/σ)_max_ < 0.001
124 parameters	Δρ_max_ = 0.97 e Å^−^^3^
0 restraints	Δρ_min_ = −1.62 e Å^−^^3^

### Special details

**Table:**

Geometry. All e.s.d.'s (except the e.s.d. in the dihedral angle between two l.s. planes) are estimated using the full covariance matrix. The cell e.s.d.'s are taken into account individually in the estimation of e.s.d.'s in distances, angles and torsion angles; correlations between e.s.d.'s in cell parameters are only used when they are defined by crystal symmetry. An approximate (isotropic) treatment of cell e.s.d.'s is used for estimating e.s.d.'s involving l.s. planes.
Refinement. Refinement of *F*^2^ against ALL reflections. The weighted *R*-factor *wR* and goodness of fit *S* are based on *F*^2^, conventional *R*-factors *R* are based on *F*, with *F* set to zero for negative *F*^2^. The threshold expression of *F*^2^ > σ(*F*^2^) is used only for calculating *R*-factors(gt) *etc*. and is not relevant to the choice of reflections for refinement. *R*-factors based on *F*^2^ are statistically about twice as large as those based on *F*, and *R*- factors based on ALL data will be even larger.

### Fractional atomic coordinates and isotropic or equivalent isotropic displacement parameters (Å^2^)

**Table:**

	*x*	*y*	*z*	*U*_iso_*/*U*_eq_	
Sn1	1.0000	0.5000	0.5000	0.0284 (3)	
Br1	1.06913 (19)	0.61962 (8)	0.68737 (17)	0.0445 (4)	
Br2	1.03622 (19)	0.59582 (8)	0.30353 (17)	0.0447 (4)	
Br3	0.66959 (17)	0.53289 (8)	0.39554 (18)	0.0423 (4)	
Br4	0.7063 (2)	0.32003 (9)	−0.12875 (19)	0.0510 (5)	
Br5	0.2397 (2)	0.45315 (8)	0.10813 (18)	0.0483 (4)	
N1	0.3496 (13)	0.2261 (5)	0.0181 (12)	0.032 (3)	
H1	0.3148	0.1789	0.0281	0.039*	
N2	0.5374 (16)	0.1644 (6)	−0.0743 (13)	0.047 (3)	
H2A	0.6200	0.1663	−0.1066	0.056*	
H2B	0.4940	0.1190	−0.0650	0.056*	
N3	0.1518 (14)	0.2743 (6)	0.1119 (13)	0.044 (3)	
H3A	0.1211	0.2259	0.1191	0.053*	
H3B	0.1029	0.3134	0.1386	0.053*	
C1	0.4759 (17)	0.2325 (7)	−0.0381 (15)	0.034 (3)	
C2	0.5364 (17)	0.3061 (7)	−0.0492 (17)	0.036 (3)	
C3	0.4653 (17)	0.3720 (8)	−0.0099 (15)	0.040 (4)	
H3	0.5047	0.4227	−0.0212	0.048*	
C4	0.3354 (18)	0.3649 (7)	0.0466 (15)	0.034 (3)	
C5	0.2745 (17)	0.2888 (8)	0.0595 (15)	0.039 (3)	

### Atomic displacement parameters (Å^2^)

**Table:**

	*U*^11^	*U*^22^	*U*^33^	*U*^12^	*U*^13^	*U*^23^
Sn1	0.0293 (7)	0.0234 (6)	0.0365 (8)	0.0000 (5)	0.0169 (6)	−0.0002 (5)
Br1	0.0485 (9)	0.0374 (7)	0.0537 (10)	−0.0074 (7)	0.0264 (8)	−0.0159 (7)
Br2	0.0494 (9)	0.0430 (8)	0.0499 (10)	−0.0016 (7)	0.0279 (8)	0.0093 (7)
Br3	0.0271 (7)	0.0319 (7)	0.0677 (10)	0.0009 (6)	0.0180 (7)	−0.0014 (7)
Br4	0.0472 (9)	0.0493 (9)	0.0706 (11)	−0.0030 (8)	0.0381 (9)	−0.0045 (8)
Br5	0.0480 (9)	0.0333 (7)	0.0680 (11)	0.0059 (7)	0.0272 (9)	−0.0091 (7)
N1	0.037 (6)	0.018 (5)	0.055 (8)	−0.003 (5)	0.032 (6)	−0.002 (5)
N2	0.061 (9)	0.029 (6)	0.059 (8)	0.003 (6)	0.032 (7)	−0.011 (6)
N3	0.042 (7)	0.035 (6)	0.069 (9)	−0.014 (6)	0.037 (7)	−0.017 (6)
C1	0.035 (8)	0.024 (6)	0.042 (9)	0.007 (6)	0.012 (7)	−0.002 (6)
C2	0.037 (8)	0.016 (6)	0.063 (9)	0.004 (6)	0.028 (7)	0.001 (6)
C3	0.038 (9)	0.032 (7)	0.048 (9)	−0.018 (7)	0.015 (8)	0.007 (6)
C4	0.049 (9)	0.017 (6)	0.034 (8)	−0.002 (6)	0.013 (7)	−0.004 (5)
C5	0.032 (8)	0.046 (8)	0.030 (8)	−0.003 (7)	0.004 (6)	−0.012 (6)

### Geometric parameters (Å, °)

**Table:**

Sn1—Br1	2.6002 (13)	N3—H3A	0.8600
Sn1—Br1^i^	2.6002 (13)	N3—H3B	0.8600
Sn1—Br2	2.5768 (14)	C1—N1	1.362 (16)
Sn1—Br2^i^	2.5768 (14)	C1—N2	1.349 (15)
Sn1—Br3	2.6131 (14)	C1—C2	1.350 (17)
Sn1—Br3^i^	2.6131 (14)	C2—Br4	1.868 (12)
N1—C5	1.357 (15)	C2—C3	1.372 (18)
N1—H1	0.8600	C3—C4	1.392 (18)
N2—H2A	0.8600	C3—H3	0.9300
N2—H2B	0.8600	C4—Br5	1.878 (12)
N3—C5	1.328 (16)	C4—C5	1.394 (18)
			
Br1—Sn1—Br1^i^	180.0	H2A—N2—H2B	120.0
Br1^i^—Sn1—Br3	88.56 (5)	C5—N3—H3A	120.0
Br1—Sn1—Br3^i^	88.56 (5)	C5—N3—H3B	120.0
Br1—Sn1—Br3	91.44 (5)	H3A—N3—H3B	120.0
Br1^i^—Sn1—Br3^i^	91.44 (5)	N2—C1—C2	123.9 (12)
Br2—Sn1—Br1	88.21 (5)	N2—C1—N1	117.8 (11)
Br2^i^—Sn1—Br1^i^	88.21 (5)	C2—C1—N1	118.3 (10)
Br2^i^—Sn1—Br1	91.79 (5)	C1—C2—Br4	120.9 (9)
Br2—Sn1—Br1^i^	91.79 (5)	C1—C2—C3	119.7 (11)
Br2^i^—Sn1—Br2	180.0	C3—C2—Br4	119.3 (9)
Br2—Sn1—Br3	89.60 (5)	C2—C3—C4	121.6 (11)
Br2^i^—Sn1—Br3^i^	89.61 (5)	C2—C3—H3	119.2
Br2^i^—Sn1—Br3	90.39 (5)	C4—C3—H3	119.2
Br2—Sn1—Br3^i^	90.39 (5)	C3—C4—Br5	123.1 (9)
Br3^i^—Sn1—Br3	180.0	C3—C4—C5	118.6 (11)
C1—N1—H1	117.6	C5—C4—Br5	118.3 (10)
C5—N1—C1	124.9 (10)	N1—C5—C4	116.9 (12)
C5—N1—H1	117.6	N3—C5—N1	118.8 (12)
C1—N2—H2A	120.0	N3—C5—C4	124.2 (12)
C1—N2—H2B	120.0		
			
N2—C1—N1—C5	−179.9 (12)	C2—C3—C4—C5	−2(2)
C2—C1—N1—C5	2(2)	C2—C3—C4—Br5	177.8 (11)
N2—C1—C2—C3	179.9 (14)	C1—N1—C5—N3	179.6 (13)
N1—C1—C2—C3	−3(2)	C1—N1—C5—C4	−2(2)
N2—C1—C2—Br4	4(2)	C3—C4—C5—N3	179.8 (13)
N1—C1—C2—Br4	−178.7 (10)	Br5—C4—C5—N3	0.4 (19)
C1—C2—C3—C4	2(2)	C3—C4—C5—N1	1.2 (19)
Br4—C2—C3—C4	178.5 (11)	Br5—C4—C5—N1	−178.2 (9)

Symmetry codes: (i) −*x*+2, −*y*+1, −*z*+1.

### Hydrogen-bond geometry (Å, °)

**Table:**

*D*—H···*A*	*D*—H	H···*A*	*D*···*A*	*D*—H···*A*
N1—H1···Br3^ii^	0.86	2.54	3.354 (9)	159
N2—H2B···Br3^ii^	0.86	2.88	3.612 (12)	144
N3—H3A···Br2^ii^	0.86	2.79	3.608 (10)	160
N3—H3B···Br1^iii^	0.86	2.82	3.604 (10)	153

Symmetry codes: (ii) −*x*+1, *y*−1/2, −*z*+1/2; (iii) −*x*+1, −*y*+1, −*z*+1.
